# Characterization of Innate Immune Responses to House Dust Mite Allergens: Pitfalls and Limitations

**DOI:** 10.3389/falgy.2021.662378

**Published:** 2021-03-11

**Authors:** Alain Jacquet

**Affiliations:** Center of Excellence in Vaccine Research and Development, Faculty of Medicine, Chulalongkorn University, Bangkok, Thailand

**Keywords:** house dust mite, allergen, innate immunity, epithelium, protease

## Abstract

Whereas house dust mite (HDM) allergy results from a dysregulated Th2-biased adaptive immune response, activation of innate immune signaling pathways is a critical prerequisite for the initiation of HDM sensitizations. Such innate sensing is mainly controlled by the airway epithelium and the skin. The resulting release of epithelial-derived proinflammatory cytokines and innate alarmins such as GM-CSF, IL-25, IL-33 and TSLP mediates the activation of ILC2 cells and cDCs to promote Th2-biased inflammation. Significant progress in the elucidation of HDM innate immune activation has been made in the past decade and highlighted key roles of the LPS/TLR4 axis, chitin-dependent pathways together with HDM protease allergens. However, the precise mechanisms by which HDM allergens are sensed by the innate immune system remain largely unknown. Such investigations are made difficult for several reasons. Among these are (1) the natural association of HDM allergens with immunostimulators from the mite exoskeleton as well as from environmental microorganisms/pollutants or endosymbiotic bacteria; (2) the purification of individual HDM allergens from extracts in sufficient amounts and devoid of any microbial and protein impurities; (3) the production of correctly folded recombinant HDM allergens which could display the same biological activity than their natural counterparts; (4) the accessibility to human epithelial samples with cellular heterogeneities and inter-donor variations; (5) the translation of experimental data from mouse models to humans is almost missing. The goal of the present mini-review is to emphasize some important limitations and pitfalls in the elucidation of innate immunostimulatory properties of HDM allergens.

## Introduction

The initiation of the HDM allergic response is dependent on skin/mucosal innate immune receptor engagement(s) to install a Pro-Th2 environment mediated by epithelial-derived proinflammatory cytokines and innate alarmins such as IL-25, IL-1b, IL-33, GM-CSF, TSLP (1). These mediators orchestrate critical cross-talks between epithelium, innate lymphoid type 2 (ILC2) and conventional dendritic (cDCs) cells to promote HDM-induced skin/airway inflammation (2).

The HDM allergome comprises at least 35 IgE-inducing allergen groups and consolidated allergenicity studies evidenced the serodominance of mite allergen groups 1, 2, and 23 (3, 4). In contrast, the hierarchy of HDM allergens capable to activate innate immune signaling pathways remains to be fully elucidated. In this context, we need to hypothesize that any HDM allergen including the minor ones could participate to these events. Whereas, key roles of LPS/TLR4 axis and PAR-2/PAR-4 signaling by HDM protease allergens have been evidenced, the complete elucidation of the innate immune mechanisms triggered by HDM allergens is a grail difficult to achieve (5). One of the main reasons is that HDM allergens are naturally associated with multiple innate immune activators as chitin from the mite exoskeleton, endosymbiotic bacteria together with microbial/environmental (bacteria, fungi, pollutants, viruses, microparticles) compounds present in the house dust.

The goal of the present narrative mini-review is to highlight the pitfalls and the limitations associated with studies evaluating innate immunomodulation by HDM allergens.

## HDM Allergenic Materials

### House Dust

HDM sensitizations are mediated by aerosolized mite feces or fragmented bodies present into the household dust. Mite fecal pellets, with a 20–30 mm average diameter (6), can reach the small airways following fragmentation as these airways are only accessible to small particles in the size range 2–6 μm (7). Analysis of the particle-size distribution of airborne Der p 2 confirmed substantial allergen association with small particles (<4.7 μm) (8). Household dust typically contains a large spectrum of environmental factors/pollutants, such as bacteria, fungal spores, particulate matter (as PM 2.5) (9, 10). All these components in association with HDM allergens, elicit innate immune activation and thus represent adjuvants to promote sensitization to HDM.

Experimental approaches based on chronic exposures of epithelial cells/animals to house dust samples are technically feasible. However, the low allergen concentration together with the presence of contaminating immunostimulators in house dust make them virtually impossible to elucidate the innate sensing of specific HDM allergens. Large differences in allergen and microbial/chemical component composition and concentration are commonly observed in house dust samples as well (9, 10). Moreover, seasonal, environmental as well as geographical variations in house dust composition negatively affect the data interpretation.

### HDM Allergen Extracts

**Figure 1:**
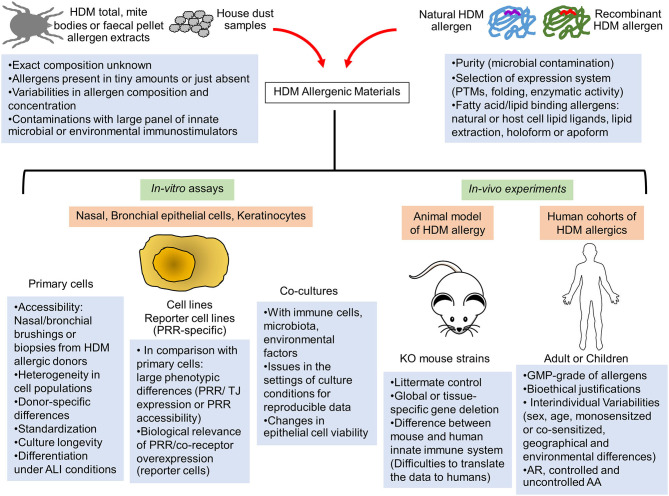
Limitations of the current *in-vitro* and *in-vivo* human and animal models to assess the innate immune activation by HDM allergens. The elucidation of innate immune receptor engagement by individual HDM allergens require reproducible batches of highly purified natural or correctly folded recombinant HDM allergens and devoid of any interfering microbial components. Interventional human studies are made difficult by interindividual variability of human innate immune response and would require GMP-grade purified HDM allergens together with bioethical justifications. Consequently, these allergenic molecules are mainly tested in mouse models of HDM-induced airway or skin inflammation or in *in-vitro* activation assays using multiple cellular systems (primary epithelial cells from HDM-allergic patients, epithelial cell lines or reporter cells expressing a specific innate sensor). Important limitations and pitfalls are associated with the experimental settings of these different assays, leading to reproducibility and data interpretation issues. AA, Allergic asthma; ALI, Air-liquid interface; AR, Allergic rhinitis; GMP, Good manufacturing practice; KO, Knockout; PRR, Pathogen recognition receptor; PTMs, Post-translational modifications; TJ, Tight Junction.

The use of commercial or in-house HDM allergen extracts provided key insights into fundamental mechanisms of HDM allergic response (1, 5). Whole mite, mite bodies or feces extracts are prepared from *in-vitro* HDM exhausted cultures. Large variabilities in allergen composition, contaminating microbial and environmental components as well as mite microbiome are observed between extracts, introducing reproducibility and data interpretation issues (11, 12) (Figure 1). A recent study evaluating several commercial HDM allergen extracts highlighted the importance to provide the lot characteristics, the concentration of major HDM allergens and LPS as well as the methods used for dosing normalization in order to compare results across different publications (13).

Proteomic analysis identified large differences between the allergen content of mite fecal pellets and mite bodies. Serodominant allergens Der p 1, Der p 2 and Der p 23 together with Der p 3, Der f 6 like allergen, Der p 9, Der p 15 and Der p 28 accumulated in mite feces mainly (3). As fecal particles penetrate more efficiently the lower lung tissues than fragmented mite bodies, mite fecal allergens must represent the first ones sensed by innate immune receptors in the lungs. Interestingly, Der p 1, 2, 5, 14 and Der f 6 like allergen were identified as the most abundant allergens in house dust, confirming that HDM allergens restricted to mite bodies as Der p 5 and Der p 14 are released from mite bodies degradation (3).

The complexity of the HDM allergen extract composition makes challenging investigations on the role to a given allergen in the activation of innate immune signals. To our knowledge, only the key roles of Der p 1, Der p 10 and Der p 13 were directly evidenced in experiments using HDM extracts treated with Der p 1-specific cysteine protease inhibitor, depleted in Der p 10 or Der p 13 or incubated with Der p 10/Der p 13-specific neutralizing antibodies, respectively (14–16).

### Purified Natural or Recombinant HDM Allergens

The use of purified natural or recombinant HDM allergens makes more accessible the elucidation of their immunomodulatory properties. Their purity is of course of paramount importance to discard any interfering contaminating activators. Recombinant allergens need to adopt the conformation of their natural counterpart which must be consistent from batch-to-batch (Figure 1). However, with the exception of the most abundant HDM allergens which can be isolated from extracts (Der p 1, Der p 2, Der p 3), comparative folding studies of natural and recombinant HDM allergens are unfeasible.

Whereas, a large collection of studies was focused mainly on the interactions of HDM protease allergens and Der p 2 with airway epithelium [reviewed in (5)], such investigations continually faced experimental issues and challenges to interpret the results as shown by the following examples from the literature.

Previously, the cysteine protease Der p 1, due to residual contamination by HDM serine protease allergens (Der p 3, Der p 6, Der p 9) after its purification, was considered by error to display dual serine/cysteine protease activities and to directly activate protease-activated receptor-2 (PAR-2), a receptor typically activated by serine but not cysteine proteases (17, 18). To study the effects of HDM protease allergens on the epithelial cells (activation, permeability or degradomics experiments), assays must be performed under serum-free conditions to prevent any proteolytic inhibition by serum proteins. Although such culture conditions mimic the airway surface liquid (ASL) composition, the cell viability and the expression of cellular proteins targeted by proteases could be affected (19). To maintain the cysteine residue of the Der p 1 active site under reducing form, Der p 1 needs to be treated with a reducing agent (as L-cysteine) prior to *in-vivo* administration or *in-vitro* cell activation. This reduction is optimal as well for the interactions with specific Der p 1 protease inhibitors (E-64 notably) (14). This procedure was not systematically adopted in studies involving HDM extracts (5). It is hypothesized that the airway redox environment, through high glutathione levels and the role of human glutathione-S-transferase pi, maximizes the Der p 1 cysteine-protease activity (20, 21).

Although natural Der p 2 could be isolated from mite allergen extracts (22), most of the Der p 2 characterization was performed with different recombinant forms of this allergen. Der p 2 displays structural homologies with myeloid differentiation factor-2 (MD-2), the LPS binding co-receptor of TLR4 (23). Recombinant Der p 2 (rDer p 2) produced in insect cells and in the presence of trace amount of LPS was capable to reconstitute TLR4 signaling in the absence of MD-2, demonstrating that Der p 2, by its capacity to interact with LPS, displays auto-adjuvant properties (24). However, a more recent study showed that rDer p 2 alone produced in yeast can cause a strong Th2-biased response in a TLR4-independent manner (25). Moreover, lipid binding assays evidenced that rDer p 2, contrary to rDer f 2, bound weakly to LPS (26, 27). In this study, rDer p 2 was produced as inclusion bodies in *E. coli*, suggesting that incomplete refolding process affects the conformation of the Der p 2 hydrophobic pocket and impairs interactions with LPS. More recent findings suggested that cholesterol, originated from dietary source of mite culture and from flakes of human skin in house dust, could represent the natural ligand of Der p 2 (28). Again, MS analysis of lipids extracted from rDer p 2 produced in *E. coli* did not evidence the presence of LPS. Taken together, the LPS binding capacity of natural Der p 2 remains to be more deeply investigated together with the identification of the endogen Der p 2 lipid ligand(s). We can hypothesize that the large Der p 2 hydrophobic pocket can accommodate lipid cargos with multiple structures. Their nature will be dependent on the expression system used for the Der p 2 production or the culture conditions for allergen extract preparation (Figure 1). We can speculate that innate activations triggered by Der p 2 are not limited to the unique Der p 2-LPS/TLR4 axis and particularly as lipid ligands can activate other PRRs as heterodimeric TLR1-TLR2 or TLR2-TLR6 (29).

Single allergen-based experimental conditions can overestimate the specific contribution of individual HDM allergens in the activation of innate sensors. Indeed, HDM allergic patients are never exposed to only a single allergen. Moreover, HDM allergen exposures could simultaneously activate identical innate immune signaling pathways, considering the functional redundancy of some HDM allergen groups (protease or lipid-binding proteins at least). Remarkably, the Prothrombinase activity of Der p 1 was shown to generate subsequently TLR4 ligands in airway epithelium resulting in TLR4-dependent ROS production whereas native Der p 2 failed to trigger ROS production (30). Consequently, Der p 1 could be top-ranked in the hierarchical classification of HDM allergens eliciting innate immune activation, raising uncertainties on the key contribution of Der p 2 in the TLR4 stimulation.

The present human protein substrates targeted by HDM protease allergens include soluble (as prothrombin, antitrypsin, elafin, SP-A, IL-33) or receptor/membrane associated molecules (occludin, ZO-1, PAR-2, CD23, CD25, CD40) (30, 31). The challenging elucidation of the complete substrate degradome of HDM proteases was surprisingly not explored up to now.

The glycan structures of HDM allergens could affect their innate immune recognition through interactions with C-type lectin Receptors (CLR) (32). Der p 1 and Der p 2 are naturally glycosylated and interact with mannose receptor and DC-SIGN, respectively (33–36). Recombinant Der p 7 produced in *P. pastoris* could bind to DC-SIGN as well but the glycosylation and the binding to CLR were not evidenced for native Der p 7 (37). The only HDM allergen showing binding activity to epithelial Dectin-1 was the structural tropomyosin Der p 10 allergen, however devoid of glycosylation (16).

Chitin, present in the mite exoskeleton as well as in the peritrophic membrane of fecal pellets displays size-dependent immunomodulatory properties. Large chitin structures (>40 μm) activated typical Th2 responses through notably the induction of epithelial-derived cytokines IL-25, TSLP and IL-33 (38). HDM allergens from group 12 (Blo t 12) and group 23 (Der p 23) display sequence homologies with chitin-binding peritrophins whereas those from groups 15 (Der p 15) and 18 (Der p 18) have some similarities with glycosyl hydrolase family 18 chitinases. Consequently, these allergens could mediate innate immune activation through chitin transport or chitin processing (degradation and release of chitin from the peritrophic membrane of inhaled fecal pellets). However, chitin binding assays with commercial chitin beads or shellfish chitin evidenced interactions with Blo t 12 only (39). The chitin binding capacity of Blo t 12 needs not only confirmation but the immunostimulatory properties of this allergen-chitin complex remain unraveled yet. It would be interesting to further investigate chitinase or chitin binding activities of these allergens using HDM-derived chitin (40).

## HDM-Induced Asthma Mouse Models or *in-vitro* Cell Activation Assays

### *In-vivo* Studies

Most of the *in-vivo* studies used conventional or conditional knock-out (KO) mice to investigate the role of a specific innate molecule in a defined HDM model of airway inflammation. A key report from Hammad's team evidenced the crucial role of TLR4 signaling in lung structural cells to initiate the HDM allergic response (41). To our knowledge, the rare *in-vivo* studies deciphering the role of unique HDM allergens were focused on protease allergens. The proteolytic activity of Der p 1 was essential for the initiation of HDM sensitization together with PAR-2 activation by serine protease allergens (15, 42–44). Although mice models are convenient, it is important to recognize their limitations to recapitulate human innate immunity (Figure 1). Notably, classical monocytes, crucial cells for innate immune responses in humans, do not respond to TLR7/8 (R848) and TLR4 (LPS) ligand in mice (45). Sequence variations in TLR4 and MD-2 between human and mice can help to explain the molecular basis of the species differences in TLR4:MD-2 response to lipid A stimulation (46).

Another key parameter affecting HDM-induced innate immune response is the constant interactions between microbiota and lung/gut mucosa (47). As microbiota composition and functions are dependent on environmental and dietary factors, co-housing, dietary control and breeding of independent mice lineages (KO, wild-type) in the same facility would minimize microbiota differences. These conditions optimize the reproducibility of the results and allow correct interpretations on the impact of innate marker deletion in such KO animal models. Another method to normalize microbiota between mouse groups is to design littermate-controlled experimental setup (48).

### *In-vitro* Cell Activation Assays

Several cellular sources of airway and skin epithelium were commonly used in *in-vitro* cell assays: primary nasal, bronchial cells or skin keratinocytes (commercial sources or isolates), epithelial cell lines or reporter cells expressing a specific Pathogen Recognition Receptor (PRR). All these cell systems offer advantages but have limitations as well (Figure 1). The airway epithelium is not made by a single type of cells. In the contrary, the core of the epithelial structure is pseudostratified and consists of basal, club, ciliated, and goblet cells. Each of these cells play unique roles in defense of the host (49). Moreover, rare cell types are also present and include among others pulmonary neuroendocrine cells and tuft cells. The barrier function of the epithelium is dependent on the formation of intercellular junctions (tight junctions [TJs], adherens junctions [AJs]) for the control the epithelial sheet integrity (50). The complexity of the airway epithelial structure clearly indicates that epithelial cell lines represent simplified biological systems for studies on the airway innate immune activation by HDM allergens, even if they offer controllable, versatile and reproducible setups. Moreover, the expression profile of PRRs and TJ proteins can greatly differ between different airway epithelial cells and do not correspond to their natural *in-vivo* expression (51). Whereas, the cell surface localization of TLR4 is important to sense LPS, this receptor can be localized intracellularly or on the basolateral side of the cells under homeostatic conditions (52). MD-2 expression is often low explaining partly the *in-vitro* hyporesponsiveness of primary epithelial cells to LPS (53). To our knowledge, whereas HDM-derived b-glucans/LPS induced cell surface expression of TLR2 and TLR4 in nasal and bronchial epithelia, respectively (54), effects of individual HDM allergens on the expression, cell-surface transfer and localization of TLR2 and TLR4 remains to be explored. The accessibility of TLR2/TLR4 (particularly on the basolateral side of epithelia) could be dependent as well on the degradation of TJ proteins by HDM protease allergens, suggesting that innate immune activation induced by HDM lipid binding proteins could be largely dependent on the initial intervention of protease allergens. The contribution of CD14, LBP, and CD36, important accessory molecules for TLR2 and TLR4 activation, in the initiation of the HDM-induced epithelial activation remains uninvestigated to this day (55).

Primary nasal or bronchial cell cultures from HDM allergic patients developing allergic rhinitis or allergic asthma is the optimal cell system to represent the complex architecture of the airway epithelium in the context of HDM allergy (56, 57). Such cell isolates can display a range of abnormalities at the level of epithelial integrity and innate immunity, commonly observed in HDM allergics (50). Moreover, it allows comparative studies on HDM component-resolved innate stimulation in relation to the donor atopic status. Unfortunately, important limitations make it difficult the use of primary epithelial cells: complex acquisition of cell samples, limited number of cells and passage, patient availability issues, cost, challenging genetic modification as well as *in-vitro* differentiation in air–liquid interface (ALI) conditions. All these issues together with the inherent biological variability between human samples affect the reproducibility of the results.

## Discussion

The direct innate immune activation by HDM allergens results from a myriad of interactions with epithelial innate sensors (Table 1). Research deciphering the intrinsic role of HDM allergens in the innate immune receptor engagement is challenging as these proteins are naturally associated with lipidergic and polysaccharide components. Despite multiple pitfalls and limitations described in this mini-review, consolidated data highlighted the key roles of HDM protease allergens, LPS and chitin to shape innate responses leading to the HDM allergic inflammation (5). In contrast, the innate sensing of lipid binding proteins and peritrophin/chitin binding protein/chitinase needs to be extensively investigated. This would allow to draw conclusion on their contributions in the initiation of the HDM allergic response. The HDM allergens from groups 2, 5, 7, 13, 14, 21, 22, 31, 35, exhibiting similarities with lipid binding protein and/or showing capacity to interact with lipid cargos, are all susceptible to promote pleiotropic effects on the innate immune system through interactions with TLR2, TLR4 or other receptors as TLR1, TLR6, RAGE, CD14, CD36 (24, 28, 58–60). Whether these proteins play redundant or unique role(s) in the innate immune activation remains to be investigated.

**Table 1:** Identified or putative innate immune activation by HDM allergens.

**Allergen**	**Biological activity**	**PRR activation/Protein target**	**Effects**	**Notes**
Der p 1	Cysteine protease	PAR-1/PAR-4/ TLR4 (Pro-thrombinase activity)MRGPRX1 and othersMR, DC-SIGN (glycosylation)?TJ proteins	Intracellular ROS, Proinflammatory cytokine productions IL-33 maturation Unknown Facilitate allergen uptake by DCs and PRR activation by other HDM allergens	Indispensable innate immune activatorComplete substrate degradome unelucidated*in-vivo* CLR activation?Interaction with sensory neurons?
Der p 2	MD-2-like fatty acid binding protein	TLR 4, TLR 2	Pro-inflammatory cytokine productions	Natural lipid ligand(s) partially identifiedLPS binding to be confirmedInteraction with other innate receptors/ co-receptors?
Der p 3	Trypsin-like serine protease	PAR-2, PAR-4TJ proteins	Pro-inflammatory cytokine productions, Mast cell activation Facilitate allergen uptake by DCs and PRR activation by other HDM allergens	Complete substrate degradome unelucidated
Der p 5	Lipid/fatty acid binding proteins	TLR2	Pro-inflammatory cytokine productions	Impossible to isolate enough natural Der p 5 to identify its lipid ligand(s). Mechanism of lipid binding unelucidated: conformational switch or hydrophobic pocket at the Der p 5 dimer interface?
Der p 7Blo t 7	Lipid/fatty acid binding proteins (LBP-like)	TLR2CLR? (glycosylated natural allergens)	Proinflammatory cytokine productions Unknown	Natural Der p 7 accessible but identification of the natural ligand(s) and glycosylations not investigated.
Der p 9	Collagenase	PAR-2TJ protein degradation	Pro-inflammatory cytokine productions Facilitate allergen uptake by DCs and PRR activation by other HDM allergens	Complete substrate degradome unelucidated
Der p 10	Tropomyosin	Dectin-1	Regulation of IL-33 release	Mechanism of binding of unglycosylated Der p 10 to Dectin-1 unelucidated
Blo t 12	Chitin binding protein	Unknown	Pro-inflammatory cytokine productions through chitin transport?	Blo t 12 homologs non-identified in *Dermatophagoides* mite species up to nowChitin binding activity to be confirmed
Der p 13Blo t 13	Cytosolic FABP	TLR2SAA	Pro-inflammatory cytokine productions Stimulation FPR2-IL-33 axis	Natural ligand unidentified
Der p 15	Chitinase	Unknown	Chitin processing? Release of allergens from fecal pellets?	Chitinase activity to be evaluatedChitin binding activity to be confirmed
Der p 18	Chitinase	Unknown	Chitin processing? Release of allergens from fecal pellets?	Chitinase activity to be evaluatedChitin binding activity to be confirmed
Der p 21	Lipid/fatty acid binding proteins	TLR2	Pro-inflammatory cytokine productions	Impossible to isolate enough natural Der p 21 to identify its lipid ligand(s). Mechanism of lipid binding unelucidated: conformational switch or hydrophobic pocket at the Der p 21 dimer interface?
Der f 22	Lipid binding protein, Der p/f 2 homolog	TLR4? TLR2?	Pro-inflammatory cytokine productions?	Similar innate immune activation than Der p 2?
Der p 23 Der f 23	Chitin binding protein	Unknown	Pro-inflammatory cytokine productions through chitin transport?	Chitin binding activity to be confirmed
Der f 31	Cofilin (actin-binding protein)	TLR2	IL-33, TSLP production, ILC2 activation	Native Der f 31 inaccessibleMode of interaction between actin-binding protein and TLR2 unknown
Der f 35	MD-2-like lipid-binding protein	TLR4?	Pro-inflammatory cytokine productions?	Similar innate immune activation than Der p 2/Der f 22?

*CLR, C-type lectin receptor; DC, dendritic cell; DC-SIGN, Dendritic Cell-Specific Intercellular adhesion molecule-3-Grabbing Non-integrin; FABP, Fatty acid binding protein; FPR2, N-formyl peptide receptor 2; ILC2, Innate lymphoid type 2 cell; LBP, LPS-binding protein; MD-2, Myeloid differentiation factor-2; MR, Mannose receptor; MRGPRX1, Mas-related G-protein coupled receptor member X1; PAR, Protease-activated receptor; PRR, Pathogen recognition receptor; ROS, Reactive oxygen species; SAA, Serum amyloid A; TJ, Tight junction; TLR, Toll-like receptor; TSLP, Thymic stromal lymphopoietin*.

Airway epithelium is continuously in interactions with the lung microbiome and alteration of lung microbiota is commonly observed in allergic asthmatics, leading to inappropriate inflammatory response (47). Moreover, lung epithelial cells and alveolar macrophages (AMs) communicate continually with each other to maintain lung homeostasis (61). Consequently, future studies should test individual HDM allergens in double or triple epithelial coculture model systems (epithelial cells and bacteria in the presence or absence of AMs) to mimic more closely the allergic nasal/lung environment (62). Nevertheless, these complex coculture models will inevitably generate challenging issues in experimental design and analysis.

Described as the first cell layers in contact with HDM allergens, airway epithelium and skin keratinocytes were considered as the primary cellular sensors of HDM allergens. However, recent findings shed a new light on the initiation of HDM allergic response. Transient receptor potential vanilloid 1 (TRPV1) expressing sensory neurons are activated by cysteine protease activity of HDM extracts and this early event is critical for the allergic skin inflammation (63). Intradermal administration of papain in mice triggered the release of substance P by TRPV1+ neurons which subsequently induces CD301b+ DC migration through Mas-related G-protein coupled receptor member A1 (MRGPRA1) (64). Nevertheless, substance P alone was unable to induce a Th2 cell response indicating that additional signal(s) mediated by alarmins/cytokines are required. Nevertheless, these results suggest that these nociceptors participate to the Der p 1-induced initiation of HDM allergic response. Additional works are required to support this hypothesis, notably through ablation or silencing of airway nociceptors in mouse model of Der p 1 sensitization (65).

With few exceptions, the direct translation of murine experimental data on innate immune activation by HDM allergens to human pathological events is missing. Notably, the association between TLR2, TLR4, PAR-2 and atopic diseases remains controversial or unknown (66). Clinical studies based on topical skin or intranasal administration of single purified allergen are not realistic as they would need, for each tested allergen, GMP-grade batch of highly purified protein. On the other hand, *ex-vivo* studies based on single-cell transcriptomics of human lung/skin tissue samples treated by HDM allergens would render feasible the spatiotemporal mapping of innate activation in airway/skin epithelial cell landscape (67).

Over the last two decades and despite the experimental issues described in this review, substantial advances have been made in the characterization of innate immune activation by HDM allergens. Future studies are needed not only to disclose all the aspects of these molecular pathways but also to demonstrate their clinical relevance.

## Author Contributions

AJ designed and wrote the first draft of the manuscript, contributed to the article, and approved the submitted version.

## Conflict of Interest

The author declares that the research was conducted in the absence of any commercial or financial relationships that could be construed as a potential conflict of interest.

## References

[1] Abu Khweek A Kim E Joldrichsen MR Amer AO Boyaka PN. Insights into mucosal innate immune responses in house dust mite-mediated allergic asthma. Front Immunol. (2020) 11:534501. 10.3389/fimmu.2020.534501 33424827 PMC7793902

[2] Deckers J De Bosscher K Lambrecht BN Hammad H. Interplay between barrier epithelial cells and dendritic cells in allergic sensitization through the lung and the skin. Immunol Rev. (2017) 278:131–44. 10.1111/imr.12542 28658557

[3] Waldron R McGowan J Gordon N McCarthy C Mitchell EB Fitzpatrick DA. Proteome and allergenome of the European house dust mite Dermatophagoides pteronyssinus. PLoS ONE. (2019) 14:e0216171. 10.1371/journal.pone.0216171 31042761 PMC6493757

[4] Becker S Schlederer T Kramer MF Haack M Vrtala S Resch Y. Real-Life study for the diagnosis of house dust mite allergy - the value of recombinant allergen-based IgE serology. Int Arch Allergy Immunol. (2016) 170:132–7. 10.1159/000447694 27505432

[5] Jacquet A Robinson C. Proteolytic, lipidergic and polysaccharide molecular recognition shape innate responses to house dust mite allergens. Allergy. (2020) 75:33–53. 10.1111/all.13940 31166610

[6] Tovey ER Chapman MD Wells CW Platts-Mills TA. The distribution of dust mite allergen in the houses of patients with asthma. Am Rev Respir Dis. (1981) 124:630–5. 7305119 10.1164/arrd.1981.124.5.630

[7] Darquenne C. Aerosol deposition in health and disease. J Aerosol Med Pulm Drug Deliv. (2012) 25:140–47. 10.1089/jamp.2011.0916 22686623 PMC3417302

[8] Custovic A Woodcock H Craven M Hassall R Hadley E Simpson A. Dust mite allergens are carried on not only large particles. Pediatr Allergy Immunol. (1999) 10:258–60. 10.1034/j.1399-3038.1999.00050.x 10678722

[9] Barberán A Dunn RR Reich BJ Pacifici K Laber EB Menninger HL. The ecology of microscopic life in household dust. Proc Biol Sci. (2015) 282:20151139. 10.1098/rspb.2015.1139 26311665 PMC4571696

[10] Wu W Jin Y Carlsten C. Inflammatory health effects of indoor and outdoor particulate matter. J Allergy Clin Immunol. (2018) 141:833–44. 10.1016/j.jaci.2017.12.981 29519450

[11] Casset A Mari A Purohit A Resch Y Weghofer M Ferrara R. Varying allergen composition and content affects the in vivo allergenic activity of commercial Dermatophagoides pteronyssinus extracts. Int Arch Allergy Immunol. (2012) 159:253–62. 10.1159/000337654 22722650 PMC4594775

[12] Klimov P Molva V Nesvorna M Pekar S Shcherbachenko E Erban T. Dynamics of the microbial community during growth of the house dust mite Dermatophagoides farinae in culture. FEMS Microbiol Ecol. (2019) 95:fiz153. 10.1093/femsec/fiz153 31584646

[13] Cyphert-Daly JM Yang Z Ingram JL Tighe RM Que LG. Physiologic response to chronic house dust mite exposure in mice is dependent on lot characteristics. J Allergy Clin Immunol. (2019) 144:1428–32. 10.1016/j.jaci.2019.07.019 31369802 PMC6842440

[14] Zhang J Chen J Zuo J Newton GK Stewart MR Perrior TR. Allergen delivery inhibitors: characterisation of potent and selective inhibitors of Der p 1 and their attenuation of airway responses to house dust mite allergens. Int J Mol Sci. (2018) 19:3166. 10.3390/ijms19103166 30326568 PMC6214017

[15] Gour N Lajoie S Smole U White M Hu D Goddard P. Dysregulated invertebrate tropomyosin-dectin-1 interaction confers susceptibility to allergic diseases. Sci Immunol. (2018) 3:eaam9841. 10.1126/sciimmunol.aam9841 29475849 PMC5956913

[16] Smole U Gour N Phelan J Hofer G Köhler C Kratzer B. Serum amyloid A is a soluble pattern recognition receptor that drives type 2 immunity. Nat Immunol. (2020) 21:756–65. 10.1038/s41590-020-0698-1 32572240 PMC9291269

[17] Hewitt CR Horton H Jones RM Pritchard DI. Heterogeneous proteolytic specificity and activity of the house dust mite proteinase allergen Der p I. Clin Exp Allergy. (1997) 27:201–7. 10.1111/j.1365-2222.1997.tb00694.x 9061221

[18] Asokananthan N Graham PT Stewart DJ Bakker AJ Eidne KA Thompson PJ. House dust mite allergens induce proinflammatory cytokines from respiratory epithelial cells: the cysteine protease allergen, Der p 1, activates protease-activated receptor (PAR)-2 and inactivates PAR-1. J Immunol. (2002) 169:4572–8. 10.4049/jimmunol.169.8.4572 12370395

[19] Rashid MU Coombs KM. Serum-reduced media impacts on cell viability and protein expression in human lung epithelial cells. J Cell Physiol. (2019) 234:7718–24. 10.1002/jcp.27890 30515823 PMC6519280

[20] Smith LJ Houston M Anderson J. Increased levels of glutathione in bronchoalveolar lavage fluid from patients with asthma. Am Rev Respir Dis. (1993) 147:1461–4. 10.1164/ajrccm/147.6_Pt_1.1461 8503557

[21] López-Rodríguez JC Manosalva J Cabrera-García JD Escribese MM Villalba M Barber D. Human glutathione-S-transferase pi potentiates the cysteine-protease activity of the Der p 1 allergen from house dust mite through a cysteine redox mechanism. Redox Biol. (2019) 26:101256. 10.1016/j.redox.2019.101256 31229842 PMC6597738

[22] Heymann PW Chapman MD Aalberse RC Fox JW Platts-Mills TA. Antigenic and structural analysis of group II allergens (Der f II and Der p II) from house dust mites (Dermatophagoides spp). J Allergy Clin Immunol. (1989) 83:1055–67. 10.1016/0091-6749(89)90447-8 2732406

[23] Derewenda U Li J Derewenda Z Dauter Z Mueller GA Rule GS. The crystal structure of a major dust mite allergen Der p 2, and its biological implications. J Mol Biol. (2002) 318:189–97. 10.1016/S0022-2836(02)00027-X 12054778

[24] Trompette A Divanovic S Visintin A Blanchard C Hegde RS Madan R. Allergenicity resulting from functional mimicry of a Toll-like receptor complex protein. Nature. (2009) 457:585–8. 10.1038/nature07548 19060881 PMC2843411

[25] Stremnitzer C Manzano-Szalai K Starkl P Willensdorfer A Schrom S Singer J. Epicutaneously applied Der p 2 induces a strong TH 2-biased antibody response in C57BL/6 mice, independent of functional TLR4. Allergy. (2014) 69:741–51. 10.1111/all.12399 24735481 PMC4023119

[26] Keber MM Gradisar H Jerala R. MD-2 and Der p 2 - a tale of two cousins or distant relatives? J Endotoxin Res. (2005) 11:186–92. 10.1179/096805105X35206 15949148

[27] Ichikawa S Takai T Yashiki T Takahashi S Okumura K Ogawa H. Lipopolysaccharide binding of the mite allergen Der f 2. Genes Cells. (2009) 14:1055–65. 10.1111/j.1365-2443.2009.01334.x 19678854

[28] Reginald K Chew FT. The major allergen Der p 2 is a cholesterol binding protein. Sci Rep. (2019) 90:1556. 10.1038/s41598-018-38313-9 30733527 PMC6367342

[29] van Bergenhenegouwen J Plantinga TS Joosten LA Netea MG Folkerts G Kraneveld AD. TLR2 & Co: a critical analysis of the complex interactions between TLR2 and coreceptors. J Leukoc Biol. (2013) 94:885–902. 10.1189/jlb.0113003 23990624

[30] Zhang J Chen J Allen-Philbey K Perera Baruhupolage C Tachie-Menson T Mangat SC. Innate generation of thrombin and intracellular oxidants in airway epithelium by allergen Der p 1. J Allergy Clin Immunol. (2016) 138:1224–27. 10.1016/j.jaci.2016.05.006 27345173 PMC5052125

[31] Chevigné A Jacquet A. Emerging roles of the protease allergen Der p 1 in house dust mite-induced airway inflammation. J Allergy Clin Immunol. (2018) 142:398–400. 10.1016/j.jaci.2018.05.027 29906529

[32] Foster AJ Bird JH Timmer MSM Stocker BL. The ligands of C-Type lectins. In: Yamasaki S, editor. C-Type Lectin Receptors in Immunity. Tokyo: Springer. (2016) p. 191. 10.1007/978-4-431-56015-9_13

[33] Al-Ghouleh A Johal R Sharquie IK Emara M Harrington H Shakib F. The glycosylation pattern of common allergens: the recognition and uptake of Der p 1 by epithelial and dendritic cells is carbohydrate dependent. PLoS ONE. (2012) 7:e33929. 10.1371/journal.pone.0033929 22479478 PMC3316510

[34] Deslée G Charbonnier AS Hammad H Angyalosi G Tillie-Leblond I Mantovani A. Involvement of the mannose receptor in the uptake of Der p 1, a major mite allergen, by human dendritic cells. J Allergy Clin Immunol. (2002) 110:763–70. 10.1067/mai.2002.129121 12417886

[35] Emara M Royer PJ Mahdavi J Shakib F Ghaemmaghami AM. Retagging identifies dendritic cell-specific intercellular adhesion molecule-3 (ICAM3)-grabbing non-integrin (DC-SIGN) protein as a novel receptor for a major allergen from house dust mite. J Biol Chem. (2012) 287:5756–63. 10.1074/jbc.M111.312520 22205703 PMC3285347

[36] Zhang Y Luo Y Li W Liu J Chen M Gu H. DC-SIGN promotes allergen uptake and activation of dendritic cells in patients with atopic dermatitis. J Dermatol Sci. (2016) 84:128–36. 10.1016/j.jdermsci.2016.08.008 27554335

[37] Tsai JJ Wang HC Chiu CL Liao EC. The effect of Dermatophagoides pteronyssinus group 7 allergen (Der p 7) on dendritic cells and its role in T cell polarization. Immunobiology. (2016) 221:1319–28. 10.1016/j.imbio.2016.04.002 27343171

[38] Van Dyken SJ Mohapatra A Nussbaum JC Molofsky AB Thornton EE Ziegler SF. Chitin activates parallel immune modules that direct distinct inflammatory responses via innate lymphoid type 2 and γδ T cells. Immunity. (2014) 40:414–24. 10.1016/j.immuni.2014.02.003 24631157 PMC4019510

[39] Zakzuk J Benedetti I Fernández-Caldas E Caraballo L. The influence of chitin on the immune response to the house dust mite allergen Blo T 12. Int Arch Allergy Immunol. (2014) 163:119–29. 10.1159/000356482 24335274

[40] Choi JP Lee SM Choi HI Kim MH Jeon SG Jang MH. House dust mite-derived chitin enhances Th2 cell response to inhaled allergens, mainly via a TNF-α-dependent pathway. Allergy Asthma Immunol Res. (2016) 8:362–74. 10.4168/aair.2016.8.4.362 27126730 PMC4853514

[41] Hammad H Chieppa M Perros F Willart MA Germain RN Lambrecht BN. House dust mite allergen induces asthma via Toll-like receptor 4 triggering of airway structural cells. Nat Med. (2009) 15:410–6. 10.1038/nm.1946 19330007 PMC2789255

[42] Gough L Campbell E Bayley D Van Heeke G Shakib F. Proteolytic activity of the house dust mite allergen Der p 1 enhances allergenicity in a mouse inhalation model. Clin Exp Allergy. (2003) 33:1159–63. 10.1046/j.1365-2222.2003.01716.x 12911793

[43] Post S Heijink IH Petersen AH de Bruin HG van Oosterhout AJ Nawijn MC. Protease-activated receptor-2 activation contributes to house dust mite-induced IgE responses in mice. PLoS ONE. (2014) 9:e91206. 10.1371/journal.pone.0091206 24651123 PMC3961228

[44] de Boer JD Van't Veer C Stroo I van der Meer AJ de Vos AF van der Zee JS. Protease-activated receptor-2 deficient mice have reduced house dust mite-evoked allergic lung inflammation. Innate Immun. (2014) 20:618–25. 10.1177/1753425913503387 24048772

[45] Bjornson-Hooper ZB Fragiadakis GK Spitzer MH Madhireddy D McIlwain D Nolan GP. A comprehensive atlas of immunological differences between humans, mice and non-human primates. bioRxiv. (2019). 10.1101/574160 PMC8962947 35359965

[46] Bryant CE Monie TP. Mice, men and the relatives: cross-species studies underpin innate immunity. Open Biol. (2012) 2:120015. 10.1098/rsob.120015 22724060 PMC3376732

[47] Barcik W Boutin RCT Sokolowska M Finlay BB. The role of lung and gut microbiota in the pathology of asthma. Immunity. (2020) 52:241–55. 10.1016/j.immuni.2020.01.007 32075727 PMC7128389

[48] McCoy KD Geuking MB Ronchi F. Gut microbiome standardization in control and experimental mice. Curr Protoc Immunol. (2017) 117:23.1.1–13. 10.1002/cpim.25 28369684

[49] Reid AT Sutanto EN Chander-Veerati P Looi K Li NF Iosifidis T. Chapter 3: Ground Zero—the Airway Epithelium. Rhinovirus Infections. London: Academic Press (2019). p. 61. 10.1016/B978-0-12-816417-4.00003-2

[50] Heijink IH Kuchibhotla VNS Roffel MP Maes T Knight DA Sayers I. Epithelial cell dysfunction, a major driver of asthma development. Allergy. (2020) 75:1902–17. 10.1111/all.14421 32460363 PMC7496351

[51] Papazian D Würtzen PA Hansen SW. Polarized airway epithelial models for immunological co-culture studies. Int Arch Allergy Immunol. (2016) 170:1–21. 10.1159/000445833 27240620

[52] Guillot L Medjane S Le-Barillec K Balloy V Danel C Chignard M. Response of human pulmonary epithelial cells to lipopolysaccharide involves Toll-like receptor 4 (TLR4)-dependent signaling pathways: evidence for an intracellular compartmentalization of TLR4. J Biol Chem. (2004) 279:2712–8. 10.1074/jbc.M305790200 14600154

[53] Jia HP Kline JN Penisten A Apicella MA Gioannini TL Weiss J. Endotoxin responsiveness of human airway epithelia is limited by low expression of MD-2. Am J Physiol Lung Cell Mol Physiol. (2004) 287:L428–37. 10.1152/ajplung.00377.2003 15121639

[54] Ryu JH Yoo JY Kim MJ Hwang SG Ahn KC Ryu JC. Distinct TLR-mediated pathways regulate house dust mite-induced allergic disease in the upper and lower airways. J Allergy Clin Immunol. (2013) 131:549–61. 10.1016/j.jaci.2012.07.050 23036747

[55] Lee CC Avalos AM Ploegh HL. Accessory molecules for Toll-like receptors and their function. Nat Rev Immunol. (2012) 12:168–79. 10.1038/nri3151 22301850 PMC3677579

[56] Bergougnan C Dittlein DC Hümmer E Riepl R Eisenbart S Böck D. Physical and immunological barrier of human primary nasal epithelial cells from non-allergic and allergic donors. World Allergy Organ J. (2020) 13:100109. 10.1016/j.waojou.2020.100109 32180893 PMC7063333

[57] Vries M Hesse L Jonker MR van den Berge M van Oosterhout AJ Heijink IH. Pim1 kinase activity preserves airway epithelial integrity upon house dust mite exposure. Am J Physiol Lung Cell Mol Physiol. (2015) 309:L1344–53. 10.1152/ajplung.00043.2015 26453516

[58] Jappe U Schwager C Schromm AB González Roldán N Stein K Heine H. Lipophilic allergens, different modes of allergen-lipid interaction and their impact on asthma and allergy. Front Immunol. (2019) 10:122. 10.3389/fimmu.2019.00122 30837983 PMC6382701

[59] Wang H Lin J Zeng L Ouyang C Ran P Yang P. Der f 31, a novel allergen from Dermatophagoides farinae, activates epithelial cells and enhances lung-resident group 2 innate lymphoid cells. Sci Rep. (2017) 7:8519. 10.1038/s41598-017-04878-0 28819104 PMC5561047

[60] Fujimura T Aki T Isobe T Matsuoka A Hayashi T Ono K. Der f 35: An MD-2-like house dust mite allergen that cross-reacts with Der f 2 and Pso o 2. Allergy. (2017) 72:1728–36. 10.1111/all.13192 28439905

[61] Bissonnette EY Lauzon-Joset JF Debley JS Ziegler SF. Cross-talk between alveolar macrophages and lung epithelial cells is essential to maintain lung homeostasis. Front Immunol. (2020) 11:583042. 10.3389/fimmu.2020.583042 33178214 PMC7593577

[62] De Rudder C Calatayud Arroyo M Lebeer S Van de Wiele T. Dual and triple epithelial coculture model systems with donor-derived microbiota and THP-1 macrophages to mimic host-microbe interactions in the human sinonasal cavities. mSphere. (2020) 5:e00916–19. 10.1128/mSphere.00916-19 31941815 PMC6968656

[63] Serhan N Basso L Sibilano R Petitfils C Meixiong J Bonnart C. House dust mites activate nociceptor-mast cell clusters to drive type 2 skin inflammation. Nat Immunol. (2019) 20:1435–43. 10.1038/s41590-019-0493-z 31591569 PMC6858877

[64] Perner C Flayer CH Zhu X Aderhold PA Dewan ZNA Voisin T. Substance P release by sensory neurons triggers dendritic cell migration and initiates the Type-2 immune response to allergens. Immunity. (2020) 53:1063–77.e7. 10.1016/j.immuni.2020.10.001 33098765 PMC7677179

[65] Talbot S Abdulnour RE Burkett PR Lee S Cronin SJ Pascal MA. Silencing nociceptor neurons reduces allergic airway inflammation. Neuron. (2015) 87:341–54. 10.1016/j.neuron.2015.06.007 26119026 PMC4506220

[66] Kirtland ME Tsitoura DC Durham SR Shamji MH. Toll-like receptor agonists as adjuvants for allergen immunotherapy. Front Immunol. (2020) 11:599083. 10.3389/fimmu.2020.599083 33281825 PMC7688745

[67] Hewitt RJ Lloyd CM. Regulation of immune responses by the airway epithelial cell landscape. Nat Rev Immunol. (2021) 13:1–16. 10.1038/s41577-020-00477-9 33442032 PMC7804588

